# A Comparison of Different Approaches to Unravel the Latent Structure within Metabolic Syndrome

**DOI:** 10.1371/journal.pone.0034410

**Published:** 2012-04-02

**Authors:** Andrew Woolston, Yu-Kang Tu, Paul D. Baxter, Mark S. Gilthorpe

**Affiliations:** Division of Biostatistics, Centre for Epidemiology and Biostatistics, University of Leeds, Leeds, United Kingdom; National University of Singapore, Singapore

## Abstract

**Background:**

Exploratory factor analysis is a commonly used statistical technique in metabolic syndrome research to uncover latent structure amongst metabolic variables. The application of factor analysis requires methodological decisions that reflect the hypothesis of the metabolic syndrome construct. These decisions often raise the complexity of the interpretation from the output. We propose two alternative techniques developed from cluster analysis which can achieve a clinically relevant structure, whilst maintaining intuitive advantages of clustering methodology.

**Methods:**

Two advanced techniques of clustering in the VARCLUS and matroid methods are discussed and implemented on a metabolic syndrome data set to analyze the structure of ten metabolic risk factors. The subjects were selected from the normative aging study based in Boston, Massachusetts. The sample included a total of 847 men aged between 21 and 81 years who provided complete data on selected risk factors during the period 1987 to 1991.

**Results:**

Four core components were identified by the clustering methods. These are labelled obesity, lipids, insulin resistance and blood pressure. The exploratory factor analysis with oblique rotation suggested an overlap of the loadings identified on the insulin resistance and obesity factors. The VARCLUS and matroid analyses separated these components and were able to demonstrate associations between individual risk factors.

**Conclusions:**

An oblique rotation can be selected to reflect the clinical concept of a single underlying syndrome, however the results are often difficult to interpret. Factor loadings must be considered along with correlations between the factors. The correlated components produced by the VARCLUS and matroid analyses are not overlapped, which allows for a simpler application of the methodologies and interpretation of the results. These techniques encourage consistency in the interpretation whilst remaining faithful to the construct under study.

## Introduction

Metabolic syndrome (MetS) defines a clustering of risk factors that act as an indicator for many chronic diseases such as kidney disease [1], [2], cardiovascular disease and type 2 diabetes mellitus [3]–[5], however the components of MetS are still controversial [6]. In recent literature, exploratory and confirmatory factor analyses have been used to test the latent structure of MetS, and regression modelling is used to test the relation between chronic diseases and MetS components [7], [8]. Whilst some exploratory analyses, such as principal component analysis (PCA) and exploratory factor analysis (EFA), can provide an insight into the structure of the data, the results are often difficult to interpret and methodological decisions are rarely justified in the application of the techniques. Discussion regarding the misuse of factor analysis in psychological research is quite common [9]–[11], however many of the same issues are rarely highlighted in the clinical and epidemiological literature.

Definitions of MetS have been proposed by a number of leading health bodies [12], [13]. Two of the most commonly accepted are those of the World Health Organization (WHO) and the National Cholesterol Education Adult Treatment Panel III (ATP III) [14]. A study by Ford et al. [15] compared the prevalence of MetS using these two definitions. In a nationally representative sample of 8,608 Americans, they found disagreement amongst 13.8% of the subjects classified as suffering from MetS when comparing these criteria. The variation in definitions highlights an uncertainty in the underlying mechanisms. This ultimately leads to confusion over the diagnosis of such a syndrome. The clustering amongst the metabolic risk factors should stimulate research into a further understanding of their inter-relationships, but the use of existing definitions should be implemented with caution [16], [17]. The study by Ford et al. further highlighted differences amongst subgroups of the population (e.g. 16.5% of African-American men were diagnosed as suffering from MetS using the ATP III criteria, whilst 24.9% were diagnosed using the WHO criteria). Evidence suggests that the form of the hypothesized syndrome is not consistent across populations [14], [18]. We require methodology that is flexible to accommodate this change, but remains able to identify biological consistency when it is present across subgroups and over time.

The clinical relevance should be the primary aim for selecting statistical methodology and deciding its application. When the conclusions of an explorative study are so heavily dependent on the application of the method, the reasoning behind each methodological decision must have a strong theoretical basis [9], [11]. The ease and speed of performing an EFA in modern statistical software has encouraged widespread use of the methodology, but this should only serve to heighten the caution adopted with the results. Despite attempts to warn against the dangers of misguided decision making in factor analysis, they are still commonly found in the literature [9], [19], [20]. There are also very few guidelines for researchers undertaking an EFA in applied research. Default software options set up by some software packages such as PCA [21] for factor extraction, the Guttman-Kaiser criterion [22], [23] to determine the number of factors to extract and the varimax (orthogonal) rotation [24] to obtain an interpretable solution, were often adopted with little or no justification to the clinical application. The same decisions as previous studies may be selected to ensure comparability, or researchers are simply ill-informed of the effects of their (potentially default) decisions [9].

For the study of MetS, the methodological decisions used in the application of EFA rarely appear to reflect the clinical hypothesis of the concept. It is the methodological decision making that is crucial to ensure that the analysis relates to the construct under study. The main restriction in an explorative MetS study is that to achieve such a structure, the complexity of the decision making increases and the interpretation of the results often becomes difficult. In this study, we discuss these methodological decisions in relation to current MetS theory and present two novel applications of clustering methodology. The aim of this study is to encourage a consistently high contextual validity (in parallel with appropriate methodological decisions in EFA), without the need to increase the complexity in the application of statistical methods. The results of the methods performed on a selection of metabolic risk factors demonstrate a promising agreement to the general structure of the construct, whilst also providing additional insights into the complex pathways present amongst the risk factors.

## Methods

### 2.1 Study Subjects

We analyzed cross-sectional data from a study by Shen et al. [25]. In short, the data was collected from 847 men aged between 21 and 81 years in the ‘Normative aging study’ (NAS). The ongoing study was based in Boston, Massachusetts and included a total of 2,280 predominantly white community-dwelling males (with a mean age of 61 years). The subjects were selected from an original 6,000 applicants who were screened at entry for existing health conditions. Those suffering from known chronic diseases, such as cardiovascular disease and type 2 diabetes mellitus, were excluded from the study. The 847 subjects selected for the application were those examined between 1987 and 1991 who provided complete data for the following covariates: fasting insulin (*Ins*), postchallenge insulin (*PCIns*), fasting glucose (*Glu*), postchallenge glucose (*PCGlu*), body mass index (*BMI*), waist/hip ratio (*WHR*), high density lipoprotein cholesterol (*HDL*), triglycerides (*Trig*), systolic blood pressure (*SBP*) and diastolic blood pressure (*DBP*). The method of data collection and the description of risk factors in the NAS have been presented in previous papers [25], [26].

### 2.2 Statistical Analysis

The analysis by Shen et al. [25] considered evidence from a range of exploratory studies to construct three hypothetical models for the structure of MetS. The evidence was gained from the use of EFA and in particular PCA. The subjective nature of factor analysis as an exploratory technique is highlighted by Shen. The series of factor structures underline the range of potential hypotheses and heuristic interpretations. Instead, a confirmatory factor analysis (CFA) is employed based on the results of the previous EFA studies and biological knowledge. The use of CFA is repeated in Shen et al. [18] to examine the structure of MetS across sex and ethnic groups, citing the conflicting and inconsistent results of EFA studies as motivation for choosing the methodology.

A ‘true’ factor analysis method (as opposed to a PCA) is based on the common factor model [27] – that assumes the observed covariation amongst the predictors is being caused by one or more latent factors. For example, in MetS data the observed variables are entered as “symptoms” exhibited by the patient. When an EFA is performed on the data, the researcher interprets that the factors extracted represent a “syndrome” as collectively they characterize some unobserved medical condition. When applying the methodology, the user must select the number of factors to retain and may specify a rotational method as a secondary step to obtain an interpretable solution. In addition, an arbitrary threshold may be applied to determine ‘significant’ loadings to interpret the meaning of the factors.

We consider an alternative explorative view provided from clustering methodology. The interpretation of observed covariates rather than abstract factors should make variable clustering techniques an attractive option in applied research. Problems associated with a ‘heuristic’ reading of components in factor analysis are simplified by considering distinct non-overlapping clusters, allowing for datasets with a large numbers of variables to be analyzed with substantially less difficulty and improved consistency [28], [29]. Hierarchical clustering allows for images to be constructed to aid with interpretation and effectively guarantees a ‘simple structure’ [30].

#### 2.2.1 The VARCLUS approach

An issue that hinders traditional cluster analysis as a technique to identifying latent structures is that the analysis is based on pair-wise dependencies. This means that underlying relationships amongst covariates may not be identified - for example, a variable *Z* can be approximated as a function of *X* and *Y*, but none of the variables is involved in a pair-wise near dependency. An alternative approach is to utilize factor analytic methods in a hierarchical clustering framework - labelled the VARCLUS approach [31]. To identify dependencies, the process builds clusters of covariates around latent components. The technique computes the first principal component of each cluster (beginning at a cluster containing all the covariates) and iteratively splits them into two separate clusters based on some pre-defined criteria. The user may suggest that if the second largest eigenvalue is greater than some given threshold value, this demonstrates evidence of an additional dimension. Alternatively, they may pre-define the number of clusters to extract based on external biological evidence. The variables are assigned to the cluster in which they demonstrate the highest squared correlation (i.e. 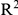) and later reassigned if the variance explained increases by including the covariate in another cluster. This approach ensures that the orthogonality assumption of PCA is relaxed. The components obtained are naturally oblique and therefore referred to as cluster components rather than principal components. This feature is beneficial to MetS study with inter-correlated clusters more likely to reflect the hypothesis of a single unified syndrome [25].

The VARCLUS process compromises on the maximal variance extraction of a PCA to maintain the intuitive advantages of clustering observed covariates. The process of directly fitting assigned labels for variables to 1 or higher dimensional clusters is labelled a ‘hard’ clustering technique. This has the advantage of retaining some of the interpretive power of a cluster analysis (in producing clusters with observed covariates), whilst making use of a components analysis to identify latent constructs within a dataset. In addition, the VARCLUS procedure in SAS provides a coefficient of determination for each variable within its own cluster (i.e. the degree to which the covariate is explained by the remaining covariates in the cluster - 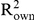) and also with the nearest cluster in which it demonstrates the greatest 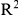 (labelled 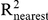). If clusters are well defined, the degree of association is maximal for variables within the same cluster and minimal to those in others. A ratio value 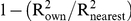 is provided to demonstrate this feature. These values are particularly useful when considered with the limitations associated with ordinary ‘hard’ (i.e. non-overlapping) clustering procedures. Whilst the clusters produced from a VARCLUS analysis are of the form of a ‘hard’ clustering method, the 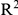 values indicate the strength of the cluster membership for each variable.

#### 2.2.2 The Matroid Approach

It is realistic for the user not to expect predictors in a complicated structure such as MetS to naturally form ‘neat’ hierarchical groups (i.e. a ‘simple’ structure); rather, we force them to be with ‘hard’ clustering techniques (such as the VARCLUS). This form of clustering is useful because of the benefits to interpretation it brings, but with it we bypass some of the subtleties in the relations that EFA attempts to identify. The VARCLUS looks to avoid this limitation by providing 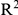 statistics. This is particularly useful as in a complicated structure, such as MetS, it would seem likely that the predictors will be involved in multiple dependencies. We propose another method in the matroid approach that could provide a compromise, whilst retaining the interpretational benefits associated with producing distinct non-overlapping clusters. Suggested by Greene [32], the method draws from existing successful ideas in the field of collinearity diagnostics and cluster analysis, whilst also introducing favourable properties of matroids, which have previously been confined largely to theoretical work.

**Figure 1:**
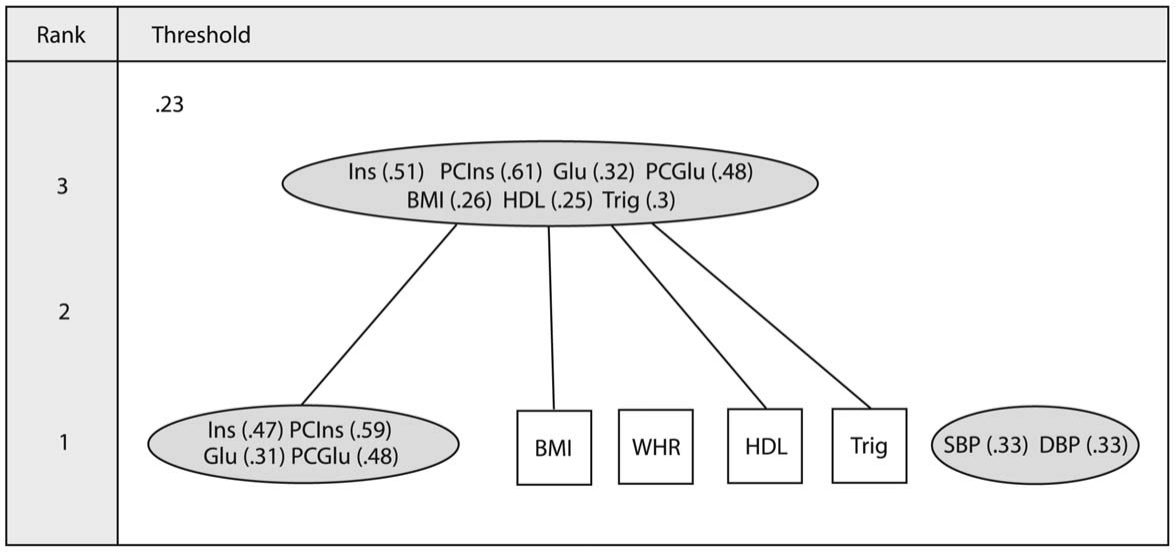
An example labelled Hasse diagram. The ellipses in the labelled Hasse diagram (LHD) demonstrate near dependencies and any variables not involved in a linear dependency are displayed as squares. The rank of each subset (illustrated on the left of the LHD) demonstrates the dimensionality of the flat. Lines between objects are used to show the sources of any dependency. An 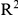 measure is displayed in brackets alongside each variable to demonstrate the fit to the flat in which it is assigned. Abbreviations: Fasting insulin (*Ins*), postchallenge insulin (*PCIns*), fasting glucose (*Glu*), postchallenge glucose (*PCGlu*), body mass index (*BMI*), waist/hip ratio (*WHR*), high density lipoprotein cholesterol (*HDL*), triglycerides (*Trig*), systolic blood pressure (*SBP*), diastolic blood pressure (*DBP*).

The matroid approach works on the collection of all subsets of variables, rather than considering the entire set at once. Initially, data are divided into all possible rearrangements of covariates and these are assigned to either a ‘dependent’ or ‘independent’ group using a suitable index. For example, a ‘dependent’ subset may be defined by the smallest eigenvalue being lower than a particular threshold. Any remaining clusters of variables are labelled ‘independent’. The group of dependent subsets are then converted into a matroid structure to ensure that they demonstrate a combinatorial arrangement corresponding to linear relationships among a collection of variables (See Welsh [33] for the axioms that define a matroid). The challenge with the matroid technique is how to convey the information of all the dependent subsets in the simplest form to the user. Greene suggests extracting a combinatorial group from those selected known as flats. A rank-*j* flat is a maximal set of covariates that can be represented by a *j*-dimensional projection [34]. In other words, if we are unable to add another covariate to the subset without increasing its rank (i.e. dimensionality), then it is labelled a flat. The flats ensure that every covariate involved in a dependency is identified. In Greene's approach we retain the general concept of ‘hard’ clustering, but we produce a dependency structure for a range of threshold values (i.e. at different strengths of dependency). A cluster is not defined only if it is uni-dimensional (as it would be for ordinary clustering), but if it exhibits a near dependency falling close to any lower dimensional subspace. The dimensionality of the flat determines its rank. A labelled Hasse diagram (LHD) is used to display the flats of the matroid (see figure 1). Each threshold produces its own hierarchical structure containing dependencies of any rank. Flats are displayed as ellipses and those variables presenting no dependency as squares (i.e. independent). The rank of each subset is illustrated on the left of the LHD and the flats joined with lines are to show the sources of any dependency. An 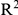 measure is displayed in brackets alongside each variable to demonstrate the fit of the variable (determined by squared correlation with the remaining covariates) to the flat in which it is assigned.

## Results

### 3.1 EFA

To gain a solution using EFA we select a principal factor analysis (PFA) method which is based on the common factor model. The intention of employing this model is to capture the clinical notion of an underlying construct amongst the manifest variables – as hypothesized by the MetS concept [6]. To determine the number of components to retain, statistical methods such as the Guttman-Kaiser criterion and parallel analysis suggest the presence of four and five factors in the model respectively. However, biological evidence should be utilized when possible to drive the analysis. Studies analysing similar risk factors of MetS (although on a different population) have proposed a four factor structure and so this will form the basis of our EFA model [35], [36]. This will also provide a direct comparison with the structures investigated in the original CFA analysis by Shen et al. An oblique ‘promax’ rotation [37] (i.e. correlated factors) is used to assess the hypothesis of a single unified MetS construct. The ‘significant’ loadings (highlighted in bold) have been selected using an arbitrary threshold of 0.3. This is suggested by Child [38] for data with sample size equal to or greater than 100 – however, this popular threshold is recommended only as a guide. Therefore, we present all the values for the benefit of the reader. The oblique ‘promax’ rotation has achieved close to a ‘simple structure’ with ‘blood pressure’ and ‘lipid’ factors clearly defined as factors 3 and 4 respectively (see table 1). Whilst the ‘blood pressure’ factor demonstrates moderate correlations with other factors (see table 2), there appears more complex inter-relationships amongst the remaining three factors. The insulin covariates (*Ins*, *PCIns*) load ‘significantly’ along with *BMI* and *WHR* on factor 1, whilst *PCIns* also loads ‘significantly’ on factor 2 along with the glucose covariates (*Glu*, *PCGlu*). There are also high correlations between factor 1 and each of the remaining factors, suggesting that obesity may be a central underlying factor of the MetS construct.

**Table 1:** The factor pattern from an exploratory factor analysis.

	Loadings				
	Factor 1	Factor 2	Factor 3	Factor 4	Communality
***Ins***	**0.70**	0.11	−0.01	−0.05	0.55
***PCIns***	**0.52**	**0.37**	0.04	−0.03	0.62
***Glu***	−0.06	**0.63**	−0.08	0.06	0.36
***PCGlu***	−0.03	**0.77**	0.04	0	0.58
***BMI***	**0.63**	−0.08	0	0.09	0.4
***WHR***	**0.53**	−0.13	0	0.12	0.29
***Trig***	−0.08	0	0.06	**−0.57**	0.36
***HDL***	0.07	0.09	0.08	**0.55**	0.41
***SBP***	−0.06	0.08	**0.69**	0.01	0.48
***DBP***	0.06	−0.11	**0.69**	−0.01	0.47

A principal factor analysis is selected with four factors retained and an oblique promax rotation used. Significance is highlighted in bold text and is determined by a factor loading >0.3. The significant loadings suggest a blood pressure factor (factor 3) and a lipid factor (factor 4). Factor 1 and factor 2 demonstrate some overlap with *PCIns* loading significantly on each. Abbreviation: Fasting insulin (*Ins*), postchallenge insulin (*PCIns*), fasting glucose (*Glu*), postchallenge glucose (*PCGlu*), body mass index (*BMI*), waist/hip ratio (*WHR*), high density lipoprotein cholesterol (*HDL*), triglycerides (*Trig*), systolic blood pressure (*SBP*), diastolic blood pressure (*DBP*).

**Table 2:** Inter-factor correlations from the exploratory factor analysis solution.

	Factor 1	Factor 2	Factor 3	Factor 4
**Factor 1**	1	0.54	0.36	0.49
**Factor 2**	0.54	1	0.27	0.27
**Factor 3**	0.36	0.27	1	0.13
**Factor 4**	0.49	0.27	0.13	1

An oblique solution produces correlated factors. The inter-factor correlations demonstrate a high correlation between factor 1 and factor 2 (0.54). There is also a large correlation between factor 1 and factor 4 (0.49).

### 3.2 VARCLUS

**Figure 2:**
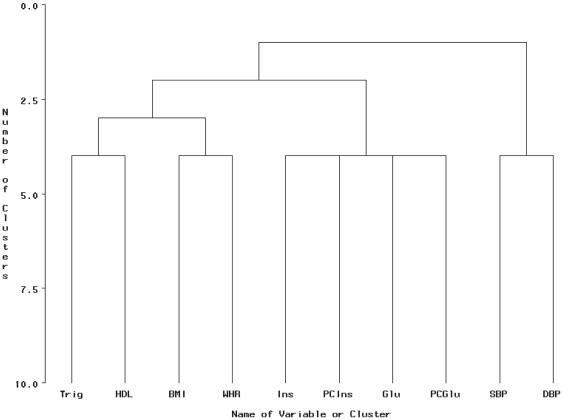
A dendrogram of the cluster structure produced by VARCLUS. A hierarchical clustering produced from the VARCLUS analysis with four cluster components selected. Abbreviation: Fasting insulin (*Ins*), postchallenge insulin (*PCIns*), fasting glucose (*Glu*), postchallenge glucose (*PCGlu*), body mass index (*BMI*), waist/hip ratio (*WHR*), high density lipoprotein cholesterol (*HDL*), triglycerides (*Trig*), systolic blood pressure (*SBP*), diastolic blood pressure (*DBP*).

The ‘PROC VARCLUS’ algorithm in SAS is an example of the VARCLUS procedure described in section 2.2.1 and has been used in the following application. To ensure comparability, we specified a maximum cluster option of four cluster components and a PCA extraction to construct latent clusters. The cluster dendrogram is illustrated in figure 2. The four cluster components listed in table 3 appear to relate to ‘lipid’ (cluster 1), ‘blood pressure’ (cluster 2), ‘insulin resistance’ (cluster 3) and ‘obesity’ (cluster 4). This is analogous to the CFA structures specified by model 1 and model 3 in Shen et al. [25]. The low 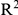 ratios 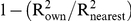 for clusters 1, 2 and 4 indicate that the cluster components are ‘well formed’. However, the ‘insulin resistance’ cluster component exhibits high values for the 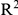 ratio for *Ins* and *Glu* risk factors. The cluster structure analysis in table 4 suggests that *Ins* in particular loads highly on the ‘obesity’ component and *BMI* similarly on the ‘insulin resistance’ component (note that ‘loadings’ demonstrate the correlation between the covariate and the cluster component). This is further evidenced by the correlation between the ‘insulin resistance’ and ‘obesity’ cluster components shown in table 5.

**Table 3:** A table of 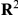 measures demonstrating the ‘quality’ of each cluster component.

	Variable	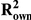	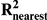	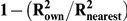
Cluster 1	***Trig***	0.735	0.076	0.287
	***HDL***	0.735	0.12	0.301
Cluster 2	***SBP***	0.785	0.058	0.228
	***DBP***	0.785	0.031	0.222
Cluster 3	***Ins***	0.542	0.208	0.579
	***PCIns***	0.704	0.149	0.348
	***Glu***	0.42	0.035	0.601
	***PCGlu***	0.624	0.044	0.394
Cluster 4	***BMI***	0.73	0.17	0.325
	***WHR***	0.73	0.087	0.296

The 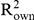 demonstrate the 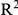 of the variable when regressed on the remaining variables in the cluster to which it is assigned. The 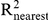 is the greatest 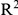 when the variable is regressed on any other cluster produced in the analysis. The 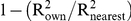 is a measure of cluster ‘quality’. When a variable has a high 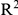 within its own cluster and low to any other, the variable demonstrates a strong fit to the cluster in whch it is assigned. Abbreviation: Fasting insulin (*Ins*), postchallenge insulin (*PCIns*), fasting glucose (*Glu*), postchallenge glucose (*PCGlu*), body mass index (*BMI*), waist/hip ratio (*WHR*), high density lipoprotein cholesterol (*HDL*), triglycerides (*Trig*), systolic blood pressure (*SBP*), diastolic blood pressure (*DBP*).

**Table 4:** The correlation (or loading) between each covariate and the cluster components.

	Cluster 1	Cluster 2	Cluster 3	Cluster 4
***Ins***	−0.297	0.192	**0.736**	0.456
***PCIns***	−0.332	0.226	**0.839**	0.386
***Glu***	−0.169	0.051	**0.648**	0.187
***PCGlu***	−0.187	0.169	**0.79**	0.211
***BMI***	−0.297	0.164	0.412	**0.854**
***WHR***	−0.268	0.13	0.295	**0.854**
***Trig***	**0.857**	−0.017	−0.219	−0.275
***HDL***	**−0.857**	0.152	0.346	0.293
***SBP***	−0.099	**0.886**	0.24	0.129
***DBP***	−0.076	**0.886**	0.145	0.176

The loadings produced in a VARCLUS analysis are analogous to factor loadings in a factor analysis. Each loading represents the correlation of the variable with the cluster component. The loadings of the variables assigned to the cluster component are highlighted in bold. Abbreviation: Fasting insulin (*Ins*), postchallenge insulin (*PCIns*), fasting glucose (*Glu*), postchallenge glucose (*PCGlu*), body mass index (*BMI*), waist/hip ratio (*WHR*), high density lipoprotein cholesterol (*HDL*), triglycerides (*Trig*), systolic blood pressure (*SBP*), diastolic blood pressure (*DBP*).

**Table 5:** The correlations between cluster components.

	Cluster 1	Cluster 2	Cluster 3	Cluster 4
Cluster 1	1	−0.099	−0.33	−0.331
Cluster 2	−0.099	1	0.217	0.172
Cluster 3	−0.33	0.217	1	0.414
Cluster 4	−0.331	0.172	0.414	1

The correlations between cluster components are analogous to inter-cluster correlations in a factor analysis with oblique rotation. Cluster 3 and cluster 4 demonstrate the strongest correlation (0.414), indicating an association between obesity and insulin resistance risk factors.

The cluster structure explains 68% of the total variation (see table 6) and reduces the dimension of the variables from 10 to 4. This simple example has allowed us to gain an immediate insight into the cluster structure, whilst still observing that the variables are likely to be involved in multiple mechanisms. The cluster structure and 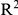 statistics indicate which covariates appear ‘least comfortable’ within the clusters and with which others they are related to. For instance, *Glu* has a high 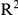 ratio (0.6), but it is not highly related to another cluster (i.e. low 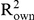) - the variable itself is not explained well by its own cluster. This adds further strength to the involvement of *Ins* and *PCIns* in other dependencies; namely a relationship between *BMI* and the insulin risk factors (as suggested in the EFA analysis). Also, *HDL* again demonstrates a high loading on the ‘insulin resistance’ cluster component. The analysis provides further evidence to the independence of the ‘blood pressure’ component (i.e. cluster 4).

**Table 6:** A summary of the variance explained by each cluster component.

	Variance Explained	Proportion Explained	2^nd^ Eigenvalue
Cluster 1	1.47	0.785	0.53
Cluster 2	1.57	0.785	0.43
Cluster 3	2.289	0.572	0.933
Cluster 4	1.46	0.73	0.54
Total variance explained:	6.789	0.679	

The 4 cluster components have explained 68% of the total variation in the data. Cluster 3 explains the largest variation in the data. The proportion explained is calculated as the total variance of the variables in the cluster divided by the variance explained. The 2^nd^ eigenvalue indicates that cluster 3 would be the next to be split if the analysis were to be extended to a 5 cluster solution.

### 3.3 The Matroid Approach

**Figure 3:**
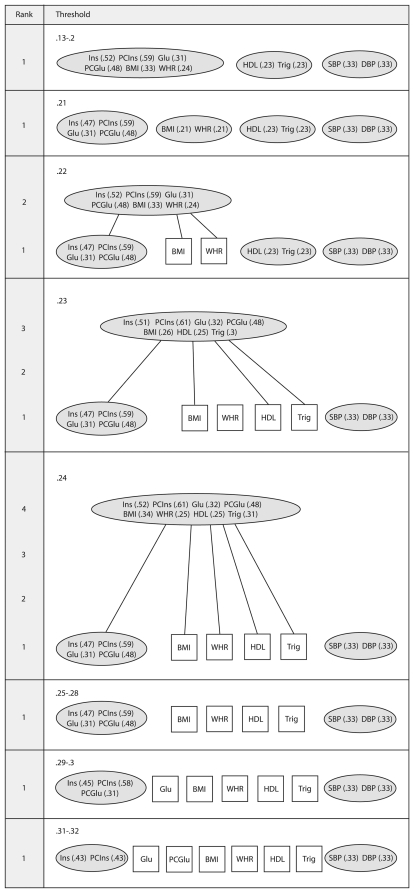
A matroid analysis of the MetS data. A labelled Hasse diagram (LHD) produced using a minimum 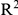 selection criteria (i.e. any subset with an 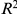 greater than the threshold value is labelled dependent). Abbreviation: Fasting insulin (*Ins*), postchallenge insulin (*PCIns*), fasting glucose (*Glu*), postchallenge glucose (*PCGlu*), body mass index (*BMI*), waist/hip ratio (*WHR*), high density lipoprotein cholesterol (*HDL*), triglycerides (*Trig*), systolic blood pressure (*SBP*), diastolic blood pressure (*DBP*).

We coded the procedure for the matroid technique using the free software package R [39]. The method has been applied to data using a criteria based on 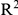; If a subset displayed an 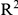 higher than the threshold value (illustrated on the left of the LHD) it is assigned dependent. The matroid LHD is displayed in figure 3. The four component structures identified by Shen et al. [25] and the VARCLUS approach are consistent with the 0.21 threshold level of the matroid depiction. In the 0.22 threshold we view an overhanging flat of rank-2 that links *BMI* and *WHR* with the insulin resistance flat. This was hypothesised in the VARCLUS example in observing a high correlation between these dependencies and a high loading of the *Ins* risk factor on the ‘obesity’ cluster component. Also, in the 0.23 threshold, *BMI*, *HDL* and *Trig* are linked with ‘insulin resistance’, however *WHR* is not. This again appears to agree with the first component of the cluster structure in the VARCLUS analysis (table 4) and the low communality estimate of the EFA (table 3). Observing the higher dimensional flats has added an intuitive description of the ‘fuzzy’ (i.e. overlapped) structure amongst the risk factors.

Perhaps the most important feature of the matroid technique is found with the higher ranked subsets extracted at particular thresholds. The intention of these is to identify subtle relationships amongst the uni-dimensional (i.e. rank-1) flats. For instance, at the 0.23 threshold, there is a rank-3 flat containing {*Ins, PCIns, Glu, PCGlu, BMI, HDL, Trig*} that is not identified elsewhere in the clustering. This may indicate a potential mechanism amongst the variables. The advantage here is that we retain the interpretive benefits of producing distinct non-overlapping clusters whilst identifying relationships potentially masked by stronger dependencies at higher thresholds. The flats in this example appear to demonstrate that the predictors (aside from *SBP*, *DBP*) are ‘fuzzy’ in nature (i.e. naturally overlapped). Overhanging dependencies of higher rank may suggest a possible hierarchical structure and could be viewed as evidence to support a concept such as MetS.

## Discussion

The methods compared in this study each provide an alternative approach to identifying and visualizing the structure of the MetS risk factors. The variability between the methods is expected as they are based on different statistical philosophies to grouping covariates. An EFA seeks to optimize the fit of the data to a common factor model in which observed variables are expressed as a *k*-dimensional collection of “common factors”, when *k* factors are retained. An oblique rotation is employed as a secondary step to provide some indication of the clustering amongst the observed variables. The identification of such clusters is in general *ad hoc* and is not incorporated into the model fitting. In comparison, the VARCLUS and matroid methods directly seek clusters of observed covariates in a single step. The role of the VARCLUS analysis is to identify 1-dimensional clusters of mutually correlated variables. The matroid approach has a similar goal, but also looks to identify higher dimensional near dependencies falling close to a lower dimensional subspace. In VARCLUS a *k*-dimensional representation can be selected by the user prior to the analysis, whilst for a matroid approach the dimensionality is selected at one of the thresholds post analysis. This selection may utilize external biological or clinical knowledge. The optimal fit of the data to common factors in an EFA (or PCA) is sacrificed for the cluster identification benefits of a VARCLUS or matroid analysis. For each of these methods the fundamental ideas have been selected to have a greater clinical relevance to the MetS hypothesis than the potentially default decisions frequently employed in an EFA.

Whilst in this study the EFA has not produced the same distinct factors as the cluster components in a VARCLUS or the flats in a matroid, the results are in agreement over the general structure of the risk factors. The oblique rotation in the EFA allows for correlation between the factors to reflect a single underlying syndrome. This correlation along with the loadings ensures that any clinical interpretation is likely to be difficult. The ‘lipid’ component (including *HDL* and *Trig*) along with the blood pressure component (including *SBP* and *DBP*) are identified in each approach. When no overlap exists (i.e. variables do not load ‘significantly’ on more than one factor) as in these factors, it is easier to interpret the correlations between the factors. The confusion in our example occurs due to the significant loading of *PCIns* on the first two factors in the EFA and the correlation between these factors. The VARCLUS and matroid methods have instead provided a direct *k*-dimensional structure for a follow up CFA if required, but also indicated how stable the clustering is. The strong association between the risk factors included in the ‘insulin resistance’ and ‘obesity’ factors are clear in each method. However, the non-overlapping clusters produced by the alternative techniques allows for a simpler interpretation of the latent variables.

The aspect that we have focussed on is the use of visual image and ‘hard’ clustering to simplify the potentially complex interpretation of an oblique solution. The difficulty with MetS is that its structure is likely to be hierarchical in nature (from a statistical perspective). A PCA with default methodological decisions is unsuitable to match the complexity or concept of this MetS construct. It may be that a hierarchical or second order factor analysis could provide an appropriate tool to analyze the structure of MetS (with the intention to separate ‘broad’ factors from ‘narrow’ factors). However, it is important to remember the context in which these methods are to be used. A likely reason that an oblique EFA or hierarchical factor analysis are rarely used in practice is due to the statistical complexity in the application and interpretation. Therefore, we remain mindful of this when promoting methodology such as VARCLUS and matroids to retain a simpler interpretation, whilst improving the consistency and appropriateness of the decision making in MetS study. This will provide a step toward the suitable level of complexity required to reflect the clinical nature of the MetS construct, without the difficulties of interpreting an EFA.

### Concluding Remarks

In this article we have concentrated on the exploratory approach. However, when combined with sound prior knowledge, a CFA can be used effectively to validate potentially complex structures; it allows for the testing of specific questions about the nature of the underlying mechanisms [40]. The use of an EFA or CFA approach should rest on the confidence of the researcher in the models used. Can we suggest a complete model, or is there sufficient uncertainty in the population structure that an explorative approach can relieve? These methods are not separate entities; they are instead a reflection of our confidence in the ‘a priori’ knowledge. As such, a considered and justified decision making process for EFA research can provide a powerful tool in developing our understanding of the MetS construct in partnership with CFA. Ideally, we would wish MetS research to favour a CFA approach, however limitations in exploratory techniques (or their application) suggest that the statistical evidence used to construct the CFA models may be less than satisfactory. The criteria for MetS, such as those proposed by the WHO and ATP III, have been developed to diagnose subjects, whereas the methods presented in this paper are not intended to form such criteria. However, the continued use of explorative techniques is of great importance. If methods such as PCA or EFA fail to reveal an underlying latent structure, the very existence of MetS becomes questionable. The intention of developing methodology such as the VARCLUS and matroid approaches is primarily to encourage consistency and reproducibility across MetS studies. It is not possible to judge from the explorative methods which will provide the ‘correct’ structure, and there may never be such a structure. Exploratory approaches should instead be valued on which yield the more useful results in terms of understanding the complex inter-relationships amongst the metabolic variables.
